# Ophiopogonin D increase apoptosis by activating p53 *via* ribosomal protein L5 and L11 and inhibiting the expression of c-Myc *via* CNOT2

**DOI:** 10.3389/fphar.2022.974468

**Published:** 2022-12-09

**Authors:** Hyun Min Ko, Wona Jee, Duckgue Lee, Hyeung-Jin Jang, Ji Hoon Jung

**Affiliations:** ^1^ College of Korean Medicine, Kyung Hee University, Seoul, South Korea; ^2^ Department of Science in Korean Medicine, Graduate School, Kyung Hee University, Seoul, China; ^3^ Soonchunhyang Institute of Medi-bio Science, Soonchunhyang University, Cheonan-si, South Korea

**Keywords:** ophiopogonin D, p53, RPL5, RPL11, apoptosis

## Abstract

Ophiopogonin D (OP-D), which is extracted from the root tuber of *Ophiopogon japonicus*, is well known for its anti-inflammatory, anti-oxidant, and anti-cancer effects. It is also therapeutic for various diseases such as diabetic myocardial injuries, obesity, atopic dermatitis, and osteoporosis. However, there are insufficient reports on the anti-cancer effects and molecular mechanisms of OP-D in colorectal cancer. Therefore, this study aimed to investigate the anti-cancer-modulating effect of OP-D on colorectal cancer. The study proved that OP-D (20–40 uM) has significant cell viability inhibition and anti-proliferative effects in Cell Counting Kit-8 (CCK-8) assay and colony formation assay. In addition, our immunofluorescence analysis data showed that OP-D (40 uM) inhibited the expression of Ki67, a cell proliferation marker, and confirmed that OP-D could induce nucleolar stress by depletion of IPO7 and XPO1. Furthermore, our western blot data showed that OP-D induced p53 expression *via* ribosomal protein (RP) L5 or L11 and inhibited c-Myc expression through CNOT2 in a dose-dependent manner. Additionally, OP-D regulated cyclin D1 and CDK4, which are well known as cell cycle regulatory proteins. OP-D consistently inhibited the phosphorylation of AKT expression in a dose-dependent manner. Furthermore, OP-D shortened c-Myc’s half-life in a time-dependent manner. Furthermore, CNOT2 knockdown enhanced the inhibitory effect of OP-D on c-Myc in colon cancer cells. Besides that, we confirmed that OP-D has a combinational anti-cancer effect of 5-FU or doxorubicin to reduce cell viability and induce apoptosis through p53 and c-Myc regulation. Altogether, our results suggest that OP-D regulates colon cancer cell proliferation and induces apoptosis by inhibiting c-Myc expression *via* activation of p53 and CNOT2 regulation. The study demonstrated that OP-D may be a promising natural anti-cancer agent for the treatment of colorectal cancer.

## Introduction

Colorectal cancer is the third deadliest and fourth most diagnosed cancer in the world (Siegel et al., 2022). Although chemotherapy, irradiation, surgery, and immunotherapy have been used for cancer therapy, recently, there have been reports that molecular target therapy is also good for regulating colorectal cancer treatment (Tariq and Ghias 2016; Nguyen and Duong 2018). Therefore, it is important to find novel genes and treatments that control cancer cells.

The tumor suppressor p53 plays an important part in various cancer cells. P53 can prevent tumor inheritance by blocking metastasis, inducing apoptosis, and stopping cell proliferation (Powell, Piwnica-Worms, and Piwnica-Worms 2014; Wawryk-Gawda et al., 2014). Various stressors such as anti-cancer reagents, depletion of ribosomal proteins and reactive oxygen species, *etc.*, can lead to increased p53 expression. P21 is a target gene of p53 and it is deeply involved in cell cycle arrest. Activation of p53 is normally related to MDM2, a major negative regulator of p53 (Moll and Petrenko 2003).

Nucleolar stress is characterized by various cellular damage-induced abnormalities in nucleolar structure and function, and activates p53 or various stress signaling pathways (Yang, Yang, and Yi 2018). Nucleolar stress is induced by a variety of factors and signals, among which it may be induced by the absence of importin 7 (IPO7) and exportin 1 (XPO1), members of the β-karyopherin family, which mediate the nuclear import of ribosomal proteins and the export of ribosomal subunits during ribosome biogenesis (Golomb et al., 2012; Zhou et al., 2012). That is, the depletion of IPO7 and XPO1 proteins impairs ribosome biosynthesis by disrupting nuclear import/export, and the resulting activation of p53 is characterized by increased RPL5 and RPL11 binding to MDM2 (Golomb, Volarevic, and Oren 2014). Therefore, nuclear stress plays an important role in apoptosis, autophagy, and cell fate (Hua et al., 2022).

c-Myc is well known to regulate cancer proliferation, angiogenesis, and apoptosis *via* the regulation of various target genes (Baudino et al., 2002; You et al., 2002; Chen et al., 2013). Many of the c-Myc target genes have been identified. c-Myc is critical for regulating the signaling pathways that facilitate cancer cell growth (Miller et al., 2012). It is reported that c-Myc activation is necessary for various essential cell processes, such as ribosomal reproduction, cell division, and survival, which are essential in cancer cell growth (Liao et al., 2014); therefore, inhibition of c-Myc activation can suppress cancer cell proliferation.

The CCR4-NOT complex (CNOT) is a regulator of mRNA stability, transcription, and translation. CNOT2 is known to regulate cancer cell proliferation, metastasis, apoptosis, angiogenesis, and autophagy (Ito et al., 2011; Jeong et al., 2017; Sohn et al., 2018). Moreover, our previous study showed that the knockdown of CNOT2 induced apoptosis by activating p53 in colon cancer cells (Jung et al., 2021). We also previously reported that the simultaneous knockdown of CNOT2 and MID1IP1 in colorectal and liver cancer cells contributes to cancer cell growth and apoptosis by significantly inhibiting c-Myc expression (Jung et al., 2020). Thus, CNOT2 is closely related to the regulation of c-Myc. MID1IP1 is an oncogene well-known as a negative regulator of AMP-activated protein kinase (AMPK), and our recent study has shown that knockdown of MID1IP1 can inhibit the expression of c-Myc in the growth of liver and colon cancer. Furthermore, Pin1 is often highly expressed in cancer cells (Chen S. et al., 2018). However, detail mechanisms for its oncogene role remain largely unknown.

*Ophiopogon* root extract is extracted from the root tuber of *Ophiopogon japonicus. Ophiopogon japonicus* is a herbal medicine for inflammation such as asthma (Chen et al., 2016). Ophiopogonin D (OP-D) is a steroidal glycoside from *Ophiopogon japonicus*. Recent studies have shown that OP-D is effective in diseases such as diabetic myocardial injuries (Li et al., 2021), obesity (Chen Y. et al., 2018), osteoporosis (Huang et al., 2015), atopic dermatitis (An et al., 2020), and has a distinct anti-oxidant and anti-inflammatory effect (Ma et al., 2018; Qiao and Jiao 2020). OP-D has also been shown to have anti-cancer effects by controlling various signaling pathways in lung cancer (Lee et al., 2018a; Lee et al., 2018b), breast cancer (Zang et al., 2016; Zhu, Wang, and Chen 2020), and prostate cancer (Lu et al., 2018; Lu et al., 2020). However, there are insufficient reports of anti-cancer effects and molecular mechanisms for OP-D in colorectal cancer. In addition, the regulation of genes such as p53, c-Myc, and CNOT2, which are mainly dealt with in this study, has not been reported in anti-cancer studies related to OP-D.

Thus, in an attempt to dissect the mechanism of OP-D, we showed that OP-D is a new anti-cancer drug for colon cancer cells. Treatment of OP-D can induce p53 expression through RPL5 or RPL11 and inhibits of c-Myc expression *via* CNOT2. OP-D also has combinational effects with 5-FU or doxorubicin has been explored in colon cancer cells.

## Materials and methods

### Cell culture and reagents

The cancer cell culture was performed following a method described previously (Jung et al., 2019; Jung et al., 2021). Briefly, HCT116^p53+/+^ cells were obtained from the American Type Culture Collection (ATCC, Manassas VA, United States), and HCT116^p53−/−^ cells were obtained by Dr. Wonchae Choe (Kyung Hee University, Seoul, Korea). All cells were cultured in RPMI-1640 medium containing 10% fetal bovine serum and 1% antibiotics at 37°C and 5% CO_2_ condition. Ophiopogonin D (OP-D) (≥98% of purity) (product ID: CFN98156) was purchased from ChemFaces (Wuhan, Hubei, China).

### RNA interference

Based on Jung’s paper, HCT116 cells were seeded in a six well plate at 7 × 10^4^ overnight. And then, transfected with p53 siRNA (Cat. No.7157-1), RPL5 siRNA (Cat. No.6125-1), RPL11 siRNA (Cat. No.6135-1), CNOT2 siRNA (Cat. No.4848-1) or control siRNA (Cat. No.SN-1003), purchased from Bioneer (Bioneer, Daejeon, Korea). The transfection assay was performed as described in Jung’s paper (Jung et al., 2021).

### Viability assay

HCT116^p53+/+^ and HCT116^p53−/−^ cells were seeded into the 96-well plate at a density of 1 × 10^4^ cells/well and incubated overnight at 37 °C with 5% CO_2_. Then, they were treated with various concentrations of OP-D for 24 h. After that, the Cell Counting Kit-8 (CCK-8) (Dojindo Molecular Technologies, Rockville, MD, United States) was distributed to each well and incubated for 1 h at 37°C with 5% CO_2_ and the absorbance of the samples was measured in a BioRad microplate reader model 680 (Biorad, Hercules, CA, United States) at 450 nm.

### Colony formation assay

HCT116^p53+/+^ cells were seeded in a 6-well plate at a density of 2 × 10^5^ cells/well, and the next day, OP-D was treated at 0, 20, or 40 uM concentrations for 24 h. Thereafter, cells treated with OP-D in a 6-well plate were re-seeded at a density of 1 × 10^4^ cells/well, respectively, and maintained at 37°C and 5% CO_2_ for 10 days until colonies were formed. Then, the Diff-Quik kit (Sysmex Corporation, Kobe, Hyogo, Japan) was used for staining the generated colony as described in a previous paper (Park, Park, and Jung 2021).

### Western blotting

Western blotting was performed as described in Jung’s paper (Jung et al., 2021). Antibodies were used for detection such as MID1IP1 (Cat No.15764-1, ProteinTech Antibody Group, Chicago, IL, United States), PARP (Cat No. 9542), RPL5 (Cat No. 14568), RPL11 (Cat No. 18163), Pin1 (Cat No. 3722), p21 (Cat No. 2947), CNOT2 (Cat No. 34214), cyclin D1 (Cat No. 2922), CDK4 (Cat No. 12790), phospho-AKT (Cat No. 4060) (Cell Signaling Technology, MA, United States), c-Myc (Cat No. 32072) (Abcam, Cambridge, United Kingdom), p53 (Cat No. sc-126) (Santa Cruz Biotechnology, Dallas, TX, United States), and β-actin (Cat No. A2228) (Sigma, St. Louis, MO, United States). Antibodies were diluted (1:500 to 1:2000) in 1-3% bovine serum albumin (BSA) and PBS-Tween20 at 4 °C overnight. All experiments were performed in triplicate, independently. The membranes were quantified by the ImageJ program.

### Immunofluorescence assay

HCT116^p53+/+^ cells were seeded on 4-well culture slides at a density of 5 × 10^4^ cells/well, and the next day, OP-D was treated with 0 or 40 uM concentration for 24 h. Then, cells were fixed with 4% paraformaldehyde for 20 min and permeabilized with 0.1% Triton X-100 for 10 min. Cells were then incubated with specific antibodies against Ki67 (Cat No. sc-23900) or IPO7 (Cat No. sc-365231) or XPO1 (Cat No. sc-7272) (Santa Cruz Biotechnology, Dallas, TX, United States) at 4 °C overnight. Cells were then incubated with Alexa Fluor 488 goat anti-mouse IgG antibody (1:500) (Invitrogen, Waltham, MA, United States) at 4°C for 2 h. Each image of stained cells was obtained according to the method of the previous paper (Ko et al., 2021).

### Protein stability assay using cycloheximide

Protein stability assay was performed as described previously (Jung et al., 2019; Jung et al., 2021). HCT116^p53+/+^ cells were treated with or without OP-D for 24 h. Cells were then treated with 50 μg/ml cycloheximide at different time points (0, 40, 80, 120 min) and then confirmed by western blotting.

### Statistical analysis

The statistical analysis was performed as described previously (Jung et al., 2019). Data were presented as means ± standard deviation (SD). One-way analysis of variance (ANOVA) followed by Tukey’s test were used to analyze the statistical significance between each group using the GraphPad Prism software (San Diego, CA, United States). Significant differences were considered if the *p*-value was less than 0.05. All experiments were conducted three times.

## Results

### OP-D reduced the viability and proliferation of HCT116 cells

**FIGURE 1:**
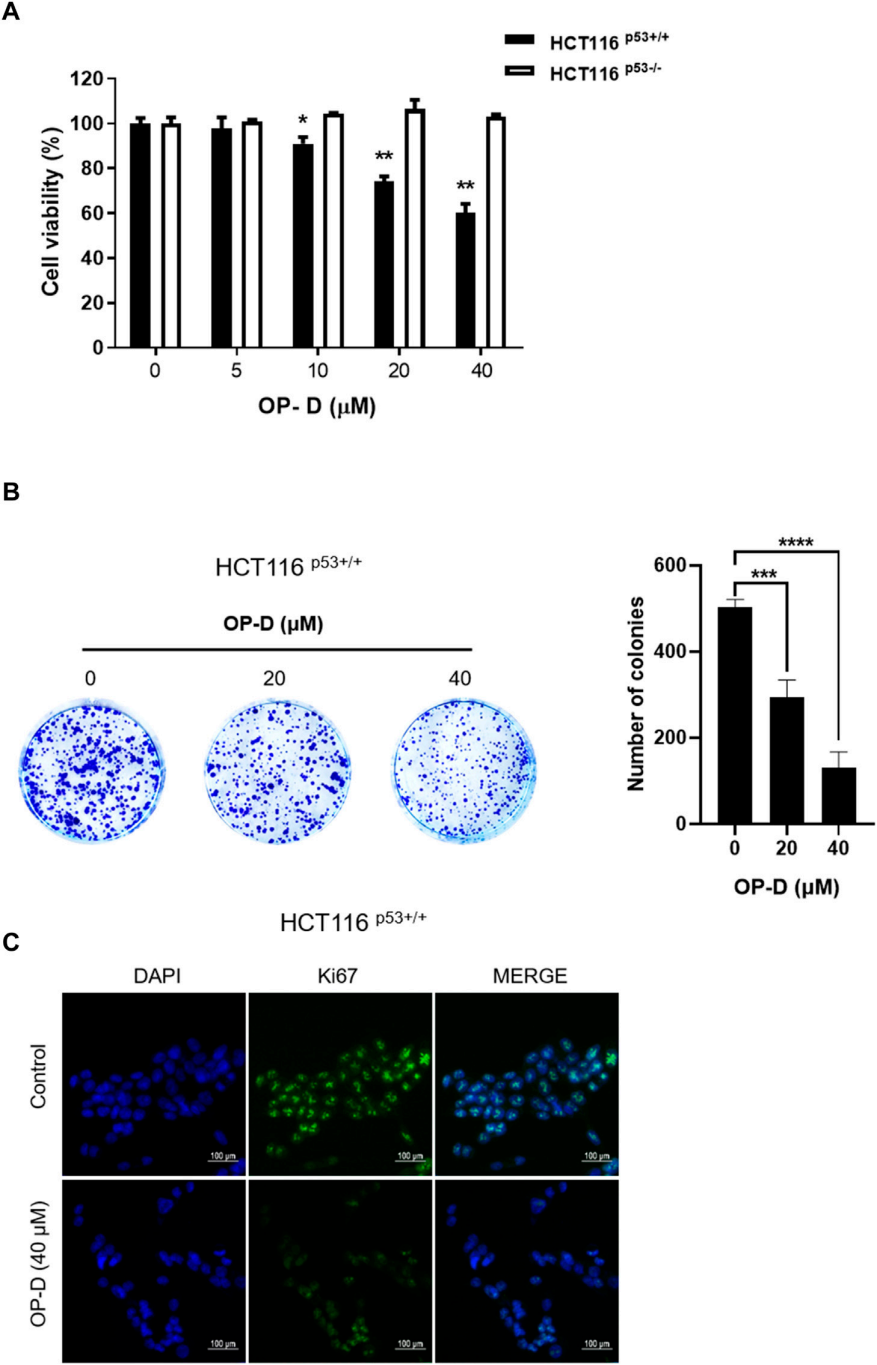
OP-D inhibited the viability and proliferation of colon cancer cells. **(A)** OP-D affects cytotoxicity in HCT116^p53+/+^ and HCT116^p53−/−^ cells. Cells were seeded in the 96 well plates and treated with 5, 10, 20, or 40 μM of OP-D for 24 h. Cytotoxicity was determined by CCK8 assay. Data represent means ± SD. *p < 0.05, **p < 0.01 *versus* untreated control. **(B)** Colony formation image (right figure) and a bar graph (left figure) in HCT116^p53+/+^cells. Data represent means ± SD. ***p < 0.005, ****p < 0.001 *versus* untreated control. **(C)** HCT116^p53+/+^cells were treated with OP-D (40 μM) for 24 h. Then, cells were fixed with 4% paraformaldehyde and stained with Ki67 antibody. Images are magnification ×200, scale bar 100 µm.

To check the cytotoxicity of OP-D in HCT116 cells, a CCK8 assay was performed in both HCT116^p53+/+^ and HCT116^p53−/−^ cells. As a result of CCK-8 analysis, OP-D significantly inhibited the cell viability of HCT116^p53+/+^ (p53 wild type) cells from a concentration of 10 uM in a dose-dependent manner. On the other hand, OP-D did not affect cell viability in HCT116^p53−/−^ cells (p53 null type) (Figure 1A). We further performed a colony formation assay associated with cell proliferation at 20, 40 uM, a concentration that clearly showed cell survival inhibitory effects at HCT116^p53++^ cells. As a result, OP-D inhibited colony formation in a dose-dependent manner (Figure 1B). In addition, we confirmed the expression of the nuclear protein Ki67, which is generally used as a marker for tumor cell proliferation, in HCT116^p53++^ cells using an immunofluorescence assay (Figure 1C). As a result, it was shown that the expression of Ki67 was remarkably suppressed by OP-D treatment (40 uM). Therefore, these data suggested that OP-D effectively reduced the cell viability and cell proliferation in HCT116^p53++^ to a greater extent than HCT116^p53−/−^ cells.

### OP-D induced p53 and its target gene p21 expression

**FIGURE 2:**
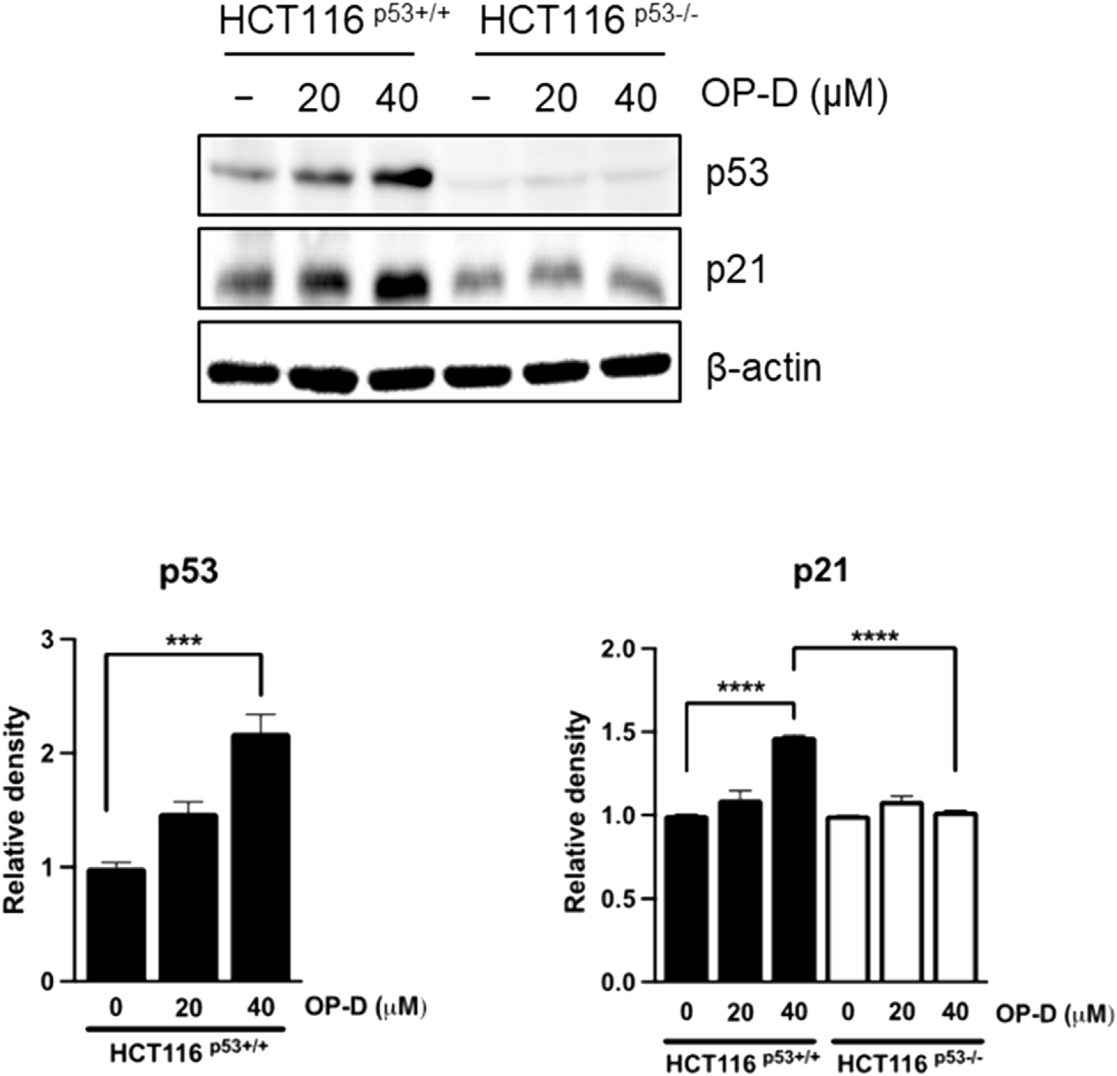
OP-D-induced p53 and its target gene p21 in HCT116^p53+/+^ and HCT116^p53−/−^ cells were treated with OP-D (0, 20, or 40 μM) for 24 h. Cell lysates were performed for p53, p21, and β-actin by Western blotting. Data represent means ± SD. ***p < 0.005, ****p < 0.001 *versus* untreated control.

p53 can induce apoptosis and stop cell proliferation (Powell, Piwnica-Worms, and Piwnica-Worms 2014; Aubrey et al., 2018). Therefore, we tested to confirm whether OP-D induced the activity of p53 and p21 (target gene of p53, and it is deeply involved in cell cycle arrest) in HCT116^p53+/+^ cells. As shown in Figure 2, OP-D induces expression of p53 and p21 dose-dependently. This result suggests that OP-D can activate p53.

### OP-D induced apoptosis by activating p53

**FIGURE 3:**
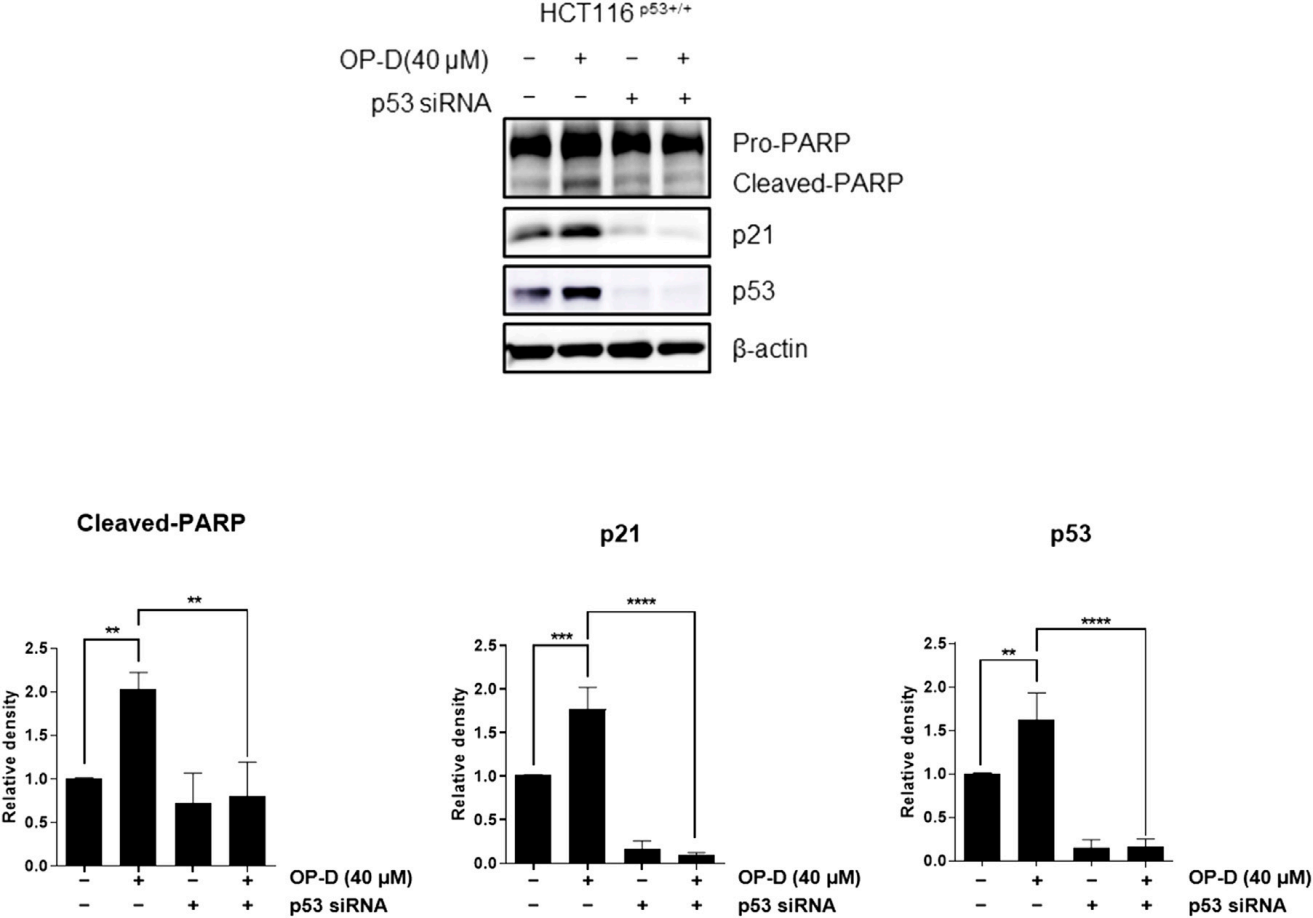
OP-D induced apoptosis through activating p53 in HCT116^p53+/+^ cells. HCT116^p53+/+^ cells were transfected with p53 siRNA for 48 h. After transfected, cells were treated with OP-D (40 μM) for 24 h. Cell lysates were performed for PARP, p21, p53, and β-actin by Western blotting. Data represent means ± SD. **p < 0.01, ***p < 0.005, ****p < 0.001.

The above results suggest that OP-D inhibits colon cancer cell viability by activating p53. An additional experiment was conducted to confirm these results. We tested whether OP-D induced apoptosis *via* activating p53. As shown in Figure 3, OP-D induced cleaved-PARP, p53, and p21; however, the knockdown of p53 by siRNA with OP-D treatment could not induce the expression of cleaved-PARP. Therefore, these results suggest that OP-D induces apoptosis by p53 activation.

**FIGURE 4:**
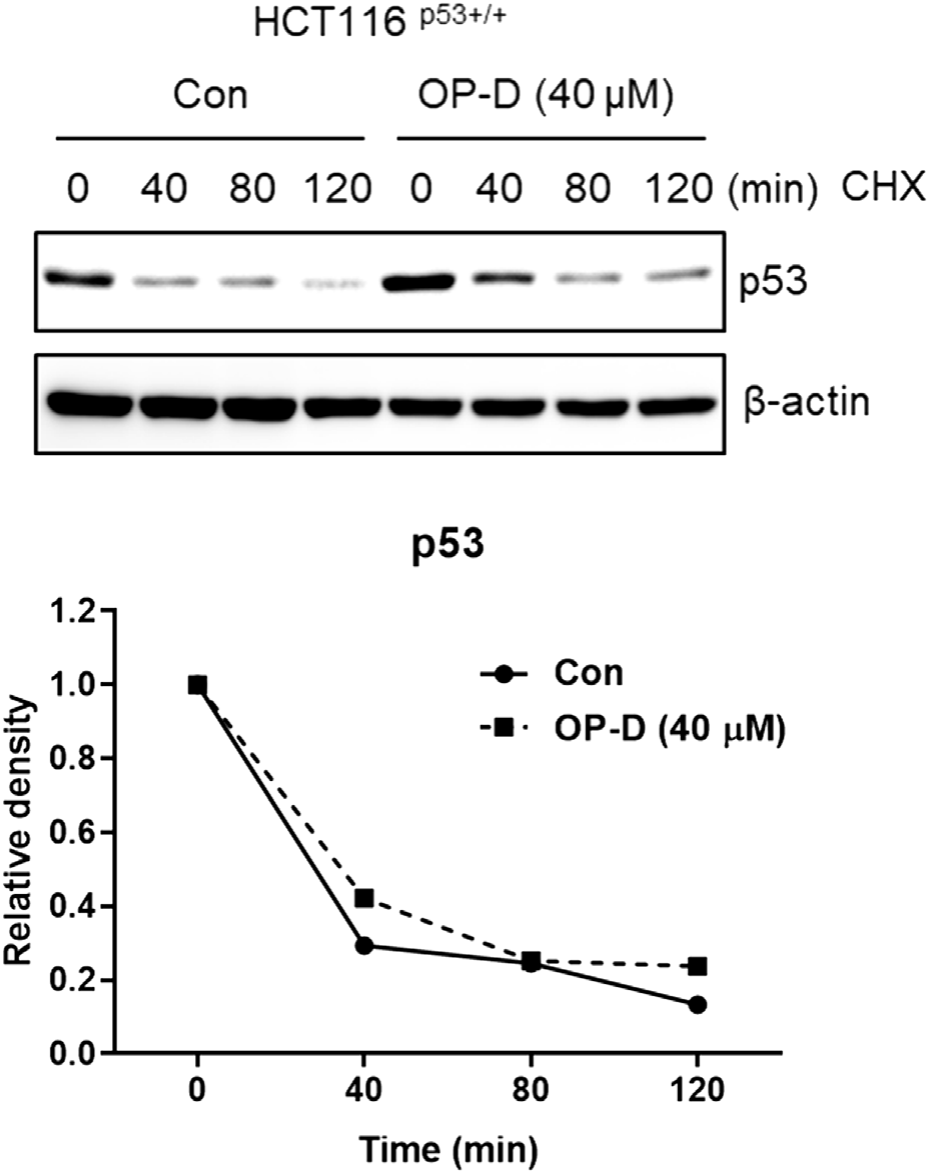
OP-D regulates c-Myc expression. HCT116^p53+/+^ cells were treated with OP-D (40 μM) for 24 h. And then, cells were exposed to 50 μg/mL of CHX at different time points. Cell lysates were performed for c-Myc and and β-actin by Western blotting.

OP-D attenuates c-Myc protein stability To check whether OP-D effects on c-Myc stability, we tested the half-life of c-Myc in HCT116^p53+/+^ cells. OP-D reduced c-Myc stability more compared to the cycloheximide only control (Figure 4).

### OP-D induces p53 *via* RPL5 or RPL11 in HCT116^p53+/+^ cells

**FIGURE 5:**
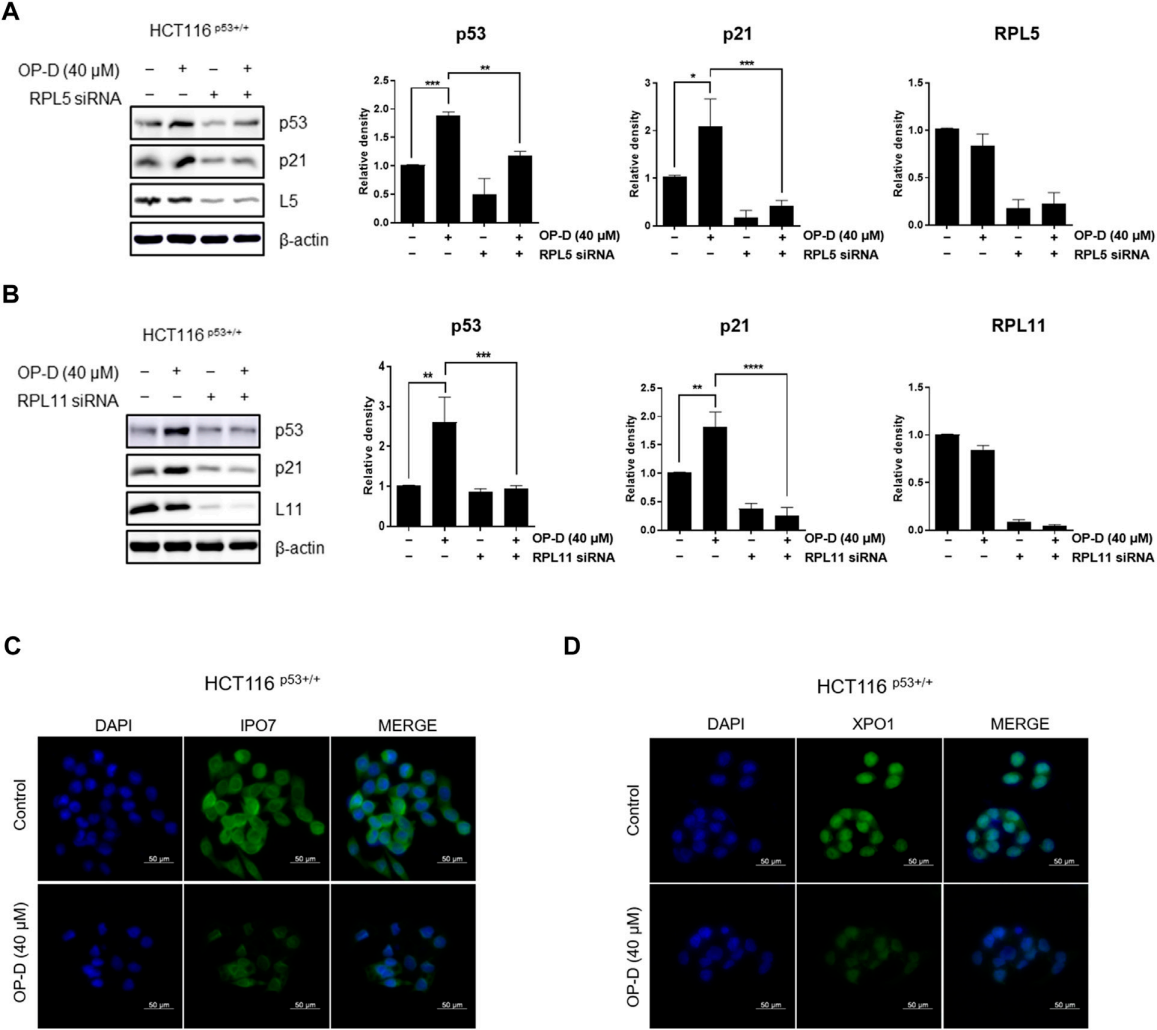
OP-D activates p53 *via* RPL5 or RPL11 by causing nucleolar stress in HCT116^p53+/+^ cells. HCT116^p53+/+^ cells were transfected with **(A)** L5 or **(B)** L11 siRNA for 48 h. After being transfected, cells were treated with OP-D (40 μM) during for 24 h. Cell lysates were performed for p53, p21, L5, L11, and β-actin by Western blotting. Data represent means ± SD. *p < 0.05, **p < 0.01, ***p < 0.005, ****p < 0.001. HCT116^p53+/+^ cells were treated with OP-D (40 μM) for 24 h. Then, cells were fixed with 4% paraformaldehyde and stained with **(C)** IPO7 or **(D)** XPO1 antibody. Images are magnification 400 ×, scale bar 50 µm.

Ribosome stress is caused by stress signals or genetic alterations that disrupt the ribosomal biogenesis in a cell. Several ribosomal proteins such as RPL5 (Lin et al., 2021), RPL11 (Bhat et al., 2004; Dai et al., 2006; Kim et al., 2021), RPL22 (Cao et al., 2017), RPL23 (Dai et al., 2004), or RPS14 (Zhou et al., 2013) are free from the ribosomes in the nucleus to the nucleoplasm where they find MDM2 to bind and inhibit MDM2 activity toward p53. Generally, RPL5, RPL11, or RPS14 bind to the central domain of MDM2 and regulate p53 activity. However, it is unclear how the RPL5 or RPL11 regulates p53 activity and it remains to be investigated. Here, we tested whether OP-D regulates p53 activity *via* RPL5 or RPL11 in HCT116^p53+/+^ cells. Interestingly, OP-D induced the expression of p53, while the knockdown of RPL5 or RPL11 could not induce p53 (Figures 5A,B). These data suggest that OP-D induced p53 *via* RPL5 or RPL11 in cancer cells.

### OP-D causes nucleolar stress *via* depletion of IPO7 and XPO1 in HCT116^p53+/+^ cells

We then analyzed the expression of IPO7 and XPO1 by immunofluorescence to determine whether OP-D induces nucleolar stress in HCT116^p53+/+^ cells. We observed that OP-D significantly suppressed the expression of IPO7 (Figure 5C) and XPO1 (Figure 5D). We thus demonstrated that OP-D treatment can induce ribosome biogenesis stress by blocking the cellular transport of ribosomal proteins and pre-ribosome required for ribosome biosynthesis through depletion of IPO7 and XPO1.

### OP-D inhibits oncogenic molecules, CNOT2, MID1IP1, and Pin1

**FIGURE 6:**
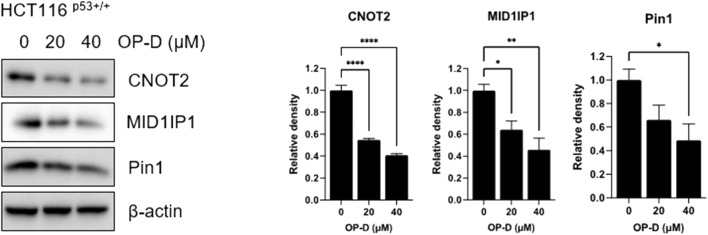
OP-D inhibited c-Myc expression in HCT116^p53+/+^ cells. HCT116^p53+/+^ cells were treated with OP-D (0, 20, or 40 μM) for 24 h. Cell lysates were performed for CNOT2, MID1IP1, Pin1 and β-actin by Western blotting. Data represent means ± SD. *p < 0.05, **p < 0.01, ****p < 0.001 versus untreated control. versus untreated control.

CNOT2, MID1IP1, and Pin1 genes are overexpressed in cancer cells of various organs, such as colorectal cancer and liver cancer, promoting tumor growth and metastasis, and commonly regulating the expression of c-Myc. Therefore, to determine whether OP-D plays an important role in oncogene regulation, we treated HCT116^p53+/+^ cells with OP-D and performed western blotting. According to the results of Figure 6, OP-D dose-dependently reduced the expression of CNOT2, MID1IP1, and Pin1 in HCT116^p53+/+^ cells, and showed a significant effect at high concentrations.

### OP-D suppressed c-Myc expression and induced apoptosis

**FIGURE 7:**
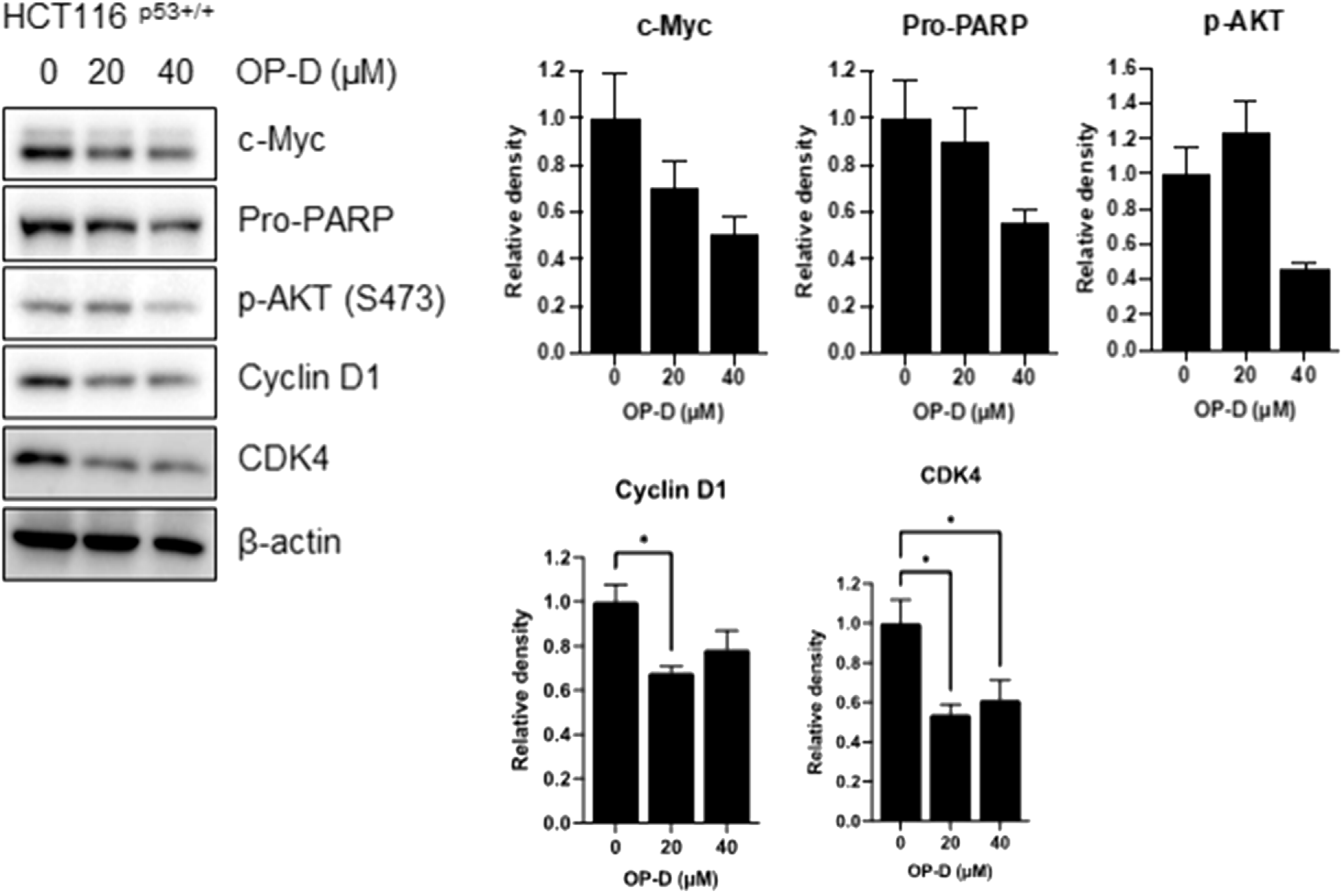
OP-D inhibited c-Myc expression in HCT116^p53+/+^ cells. HCT116^p53+/+^ cells were treated with OP-D (0, 20, or 40 μM) for 24 h. Cell lysates were performed for c-Myc, Pro-PARP, p-AKT, cyclin D1, CDK4, and β-actin by Western blotting. Data represent means ± SD. * *p* < 0.05 versus untreated control.

Although OP-D was shown to regulate various cancer cell proliferations, much more needs to be learned about its role in cancer development. To resolve this question, we first performed Western blotting to check c-Myc expression. Among oncogenic molecules, c-Myc is an oncoprotein that regulates numerous genes expression in cancer cell growth and proliferation (Dang 2012; Hsieh et al., 2015). Cancer cells with abnormally high levels of c-Myc expression proliferate without stimulating growth factors, suggesting that c-Myc can be a good target for cancer treatments. Furthermore, microRNA-145 provides a direct link between p53 and c-Myc in cancer cells (Sachdeva et al., 2009). As shown in Figure 7, OP-D reduced the c-Myc expression. Furthermore, OP-D regulates apoptotic proteins such as PARP and phosphor-AKT. We also tested the effect of OP-D on cell cycle protein markers. We found that OP-D inhibited cyclin D1 and CDK4 expression in HCT116^p53+/+^ cells.

### The role of CNOT2 in OP-D-inhibited c-Myc in colon cancer cells

**FIGURE 8:**
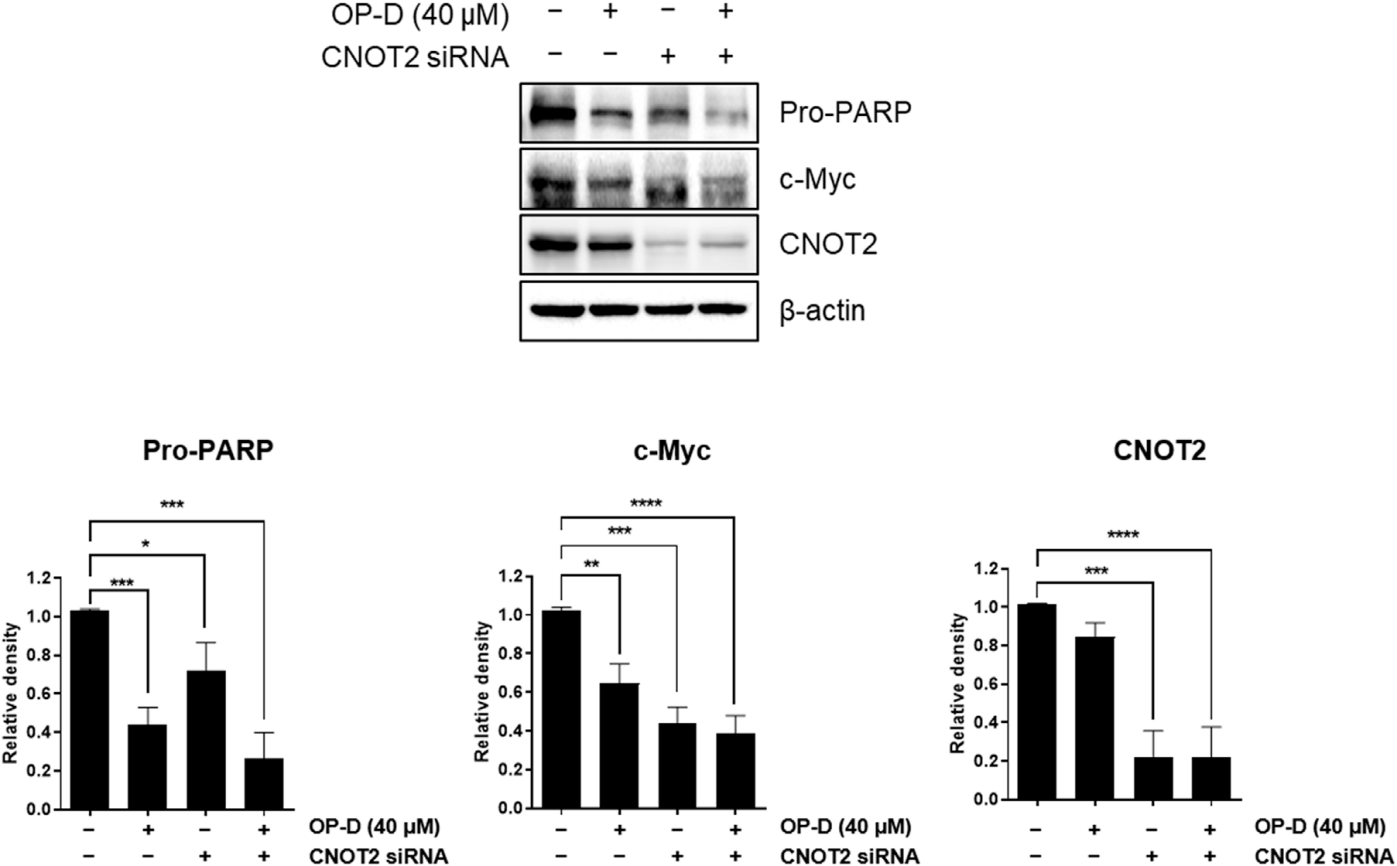
OP-D inhibits c-Myc *via* CNOT2 in HCT116^p53+/+^ cells. HCT116^p53+/+^ cells were transfected with CNOT2 siRNA or control siRNA (80 nM) for 50 h. And then, cells were treated with OP-D (0 or 40 μM) for 24 h. Cell lysates were performed for Pro-PARP, c-Myc, CNOT2, and β-actin by Western blotting. Data represent means ± SD. *p < 0.05, **p < 0.01, ***p < 0.005, *****p*p* < 0.001 versus untreated control.

Our previous paper showed that CNOT2 regulates liver cancer cell growth through c-Myc and MID1IP1. Here, CNOT2 was investigated in OP-D-suppressed c-Myc in HCT116^p53+/+^ cells. In Figure 8, CNOT2 inhibition using siRNA improved the inhibitory effect of OP-D on c-Myc expression compared with the control group. This result suggests that OP-D inhibits c-Myc expression *via* CNOT2 in colon cancer cells.

### OP-D attenuates c-Myc protein stability in colon cancer cells

**FIGURE 9:**
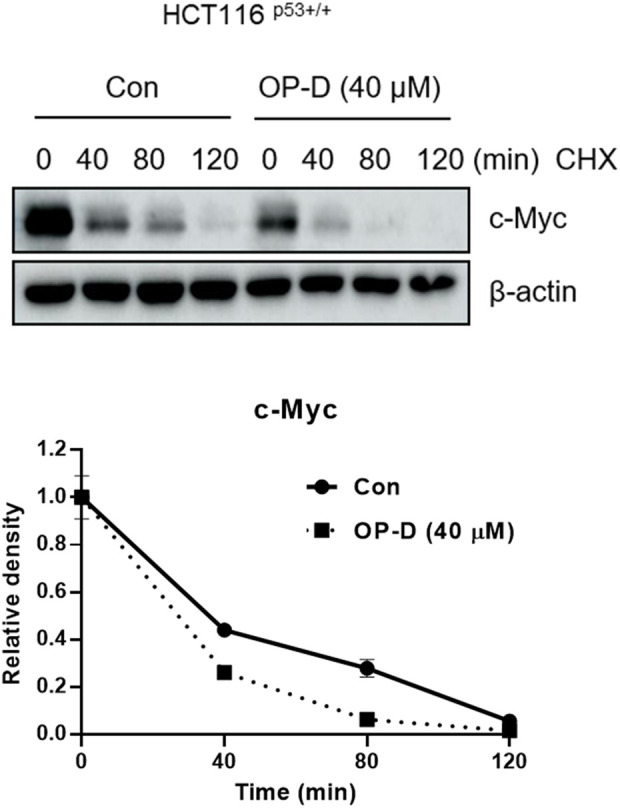
OP-D inhibits c-Myc stability in HCT116^p53+/+^ cells. HCT116^p53+/+^ cells were treated with OP-D (40 μM) for 24 h. Then, HCT116^p53+/+^ cells were cultured with cycloheximide (50 μg/ml) for 0, 40, 80, and 120 min, and then colleting the cells were collected. The cells were tested by performed by Western blotting with antibodies of c-Myc and β–actin.

To check the effect of OP-D on c-Myc stability, we confirmed the half-life of c-Myc in the absence or presence of OP-D with cycloheximide in HCT116^p53+/+^ cells. OP-D reduced c-Myc stability more compared to the cycloheximide-only in HCT116^p53+/+^ cells (Figure 9).

### Combination effect of OP-D and 5-FU or doxorubicin in colon cancer cells

**FIGURE 10:**
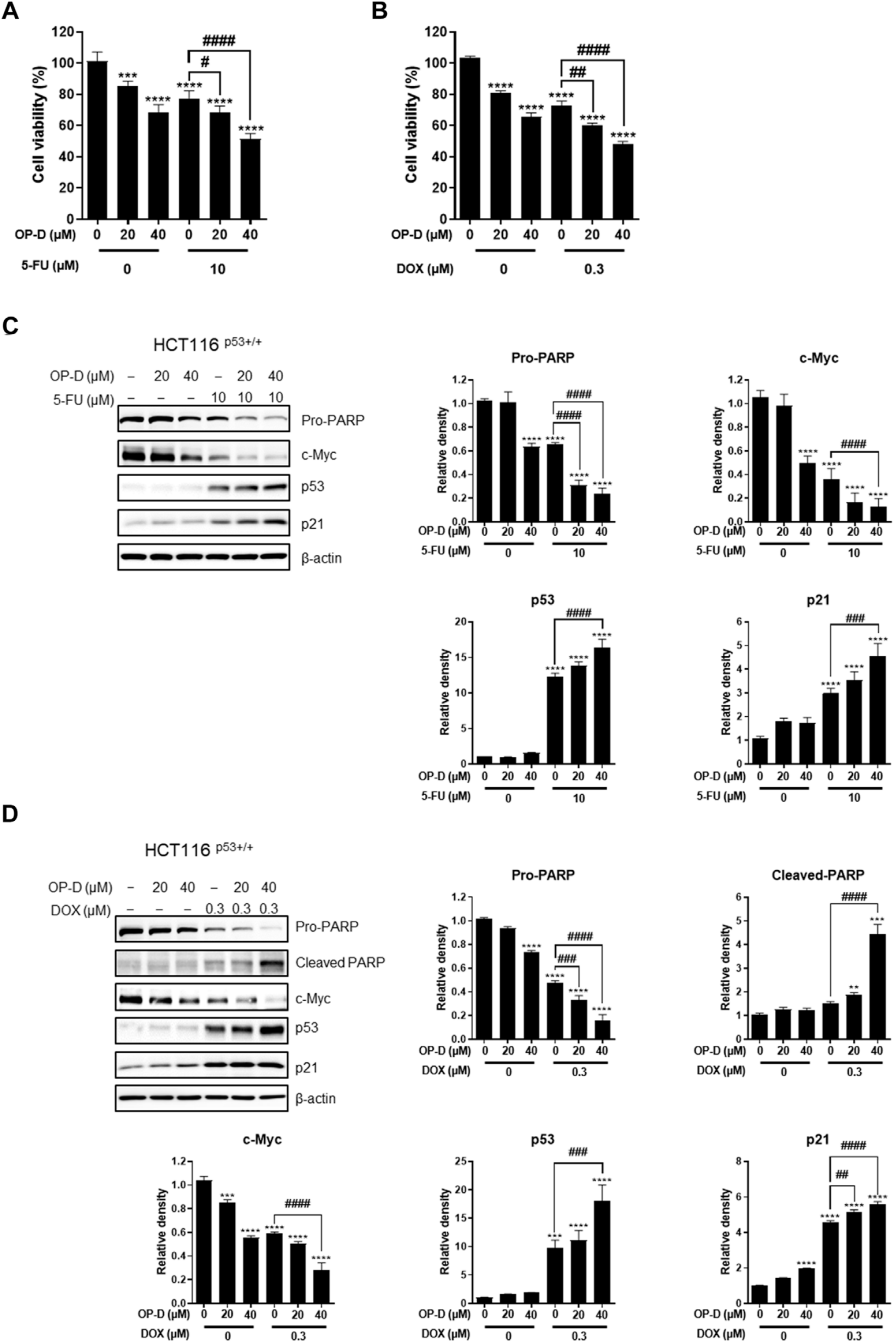
Combination effect of OP-D and 5-FU or doxorubicin in HCT116^p53+/+^ cells. **(A)** HCT116^p53+/+^ cells were treated with or without OP-D (20 or 40 μM) and 5-FU (10 μM) for 24 h. **(B)** HCT116^p53+/+^ cells were treated with or without OP-D (20 or 40 μM) and doxorubicin (0.3 μM) for 24 h. **(C)** Then, cells were collected and performed by Western blotting with various antibodies. **(D)** Then, cells were collected and performed by Western blotting with various antibodies. Data represent means ± SD. **p < 0.01, ***p < 0.005, ****p < 0.001, ##*p* < 0.01, ###*p* < 0.005, ####*p* < 0.001.

5-FU and doxorubicin have been used as treatments for colon cancer cells (Weinlander et al., 1997; Pardini et al., 2011). However, due to various side effects, the discovery and research of alternative drugs are needed. As shown in Figure 10A, the data confirmed that the growth of cancer cells was further suppressed when OP-D and 5-FU were combined. The combined treatment of OP-D and doxorubicin showed a similar result (Figure 10B). We then observed changes in protein expression in apoptosis-related factors due to combined treatment through western blotting. As shown in Figures 10 C,D, during the combined treatment, the expression of p53 and the target gene p21 was further increased, and the expression of oncogene c-Myc was further decreased.

## Discussion

Globally, colorectal cancer is one of the main causes of cancer-related deaths. Certain natural compounds are attractive as treatment options because of their lower toxicity and potential to synergize with classical anti-cancer drugs. Previously, our group reported that OP-D inhibited ERK and p38 protein expression and led to a reduction in the nuclear translocation of NF-κB (An et al., 2020). Additionally, other research groups have also published findings that OP-D regulates STAT3 in lung cancer cells (Lee, Kim, Lee, Sethi, et al., 2018). However, the underlying anti-tumor mechanism of OP-D is not fully understood. This is the first paper outlining that OP-D increases apoptosis by activating p53 *via* RPL5 and RPL11 and inhibiting c-Myc expression *via* CNOT2 in colorectal cancer cells.

First, OP-D treatment showed cytotoxic and anti-proliferative effects in colon cancer cells. Interestingly, OP-D inhibits cancer cell viability in p53 wild-type cancer cells, but not in p53-null cancer cells dose dependently. We also found that OP-D induces p53-dependent apoptosis in HCT116 cells. It is well known that p53 is a potent tumor suppressor in cancer cells (Levine 2020, 2019). Among anti-apoptotic proteins, c-Myc is known as a typical oncogene in cancers and induces cancer cell proliferation and is upregulated in various cancer cells (Chen, Liu, and Qing 2018). Interestingly, p53 and c-Myc affect each other and are involved in various cell signaling pathways in cancer cells, making them good targets for chemotherapy. Furthermore, p53 and c-Myc target genes are good targets for chemotherapy in cancer cells such as p21, PUMA, Bax, Max, etc (Dang 1999; Tokino and Nakamura 2000; Fischer 2017). Here, we showed that OP-D increases p53 expression and p21, a target gene of p53, and decreases c-Myc expression in colon cancer cells.

Ribosomal proteins are critically involved in cancer cell proliferation, cell growth, differentiation, and apoptosis in cancer cells (Liao et al., 2014; Jung et al., 2019). Ribosomal proteins also play an important role in regulating p53 expression. We, therefore, confirmed that OP-D induces apoptosis through the activation of p53 *via* RPL5 and RPL11. To further demonstrate this hypothesis, we investigated the expression of IPO7 and XPO1, the β-karyopherin gene characterized by activation of p53 dependent on RPL5 and RPL11. As a result, OP-D appears to cause nucleolar stress by inhibiting the expression of IPO7 and XPO1, and activating the expression of p53 using these and unassembled RPL5 and RPL11. Furthermore, CNOT2, a subunit of the CCR4-NOT complex, is known to be related to angiogenesis, metastasis, cell proliferation, and autophagy in many types of cancer cells. Recently, our papers showed that c-Myc was mediated by CNOT2 *via* MID1IP1 and RPL5 or RPL11 in liver cancer cells, and CNOT2 knockdown induced p53 expression in colon cancer cells (Jung et al., 2021). Through this clue, we thought that finding a new drug that regulates these oncogenes such as c-Myc, CNOT2, and tumor suppressor p53 would enable efficient cancer treatment. In testing this hypothesis, we showed that OP-D inhibits c-Myc expression *via* CNOT2. Altogether, these results indicate that OP-D induces apoptosis by activating p53 *via* RPL5 and RPL11 in colon cancer cells. Recently, the combination of commercial anti-cancer drugs such as 5-FU and doxorubicin with natural compounds is attractive because the side effects of 5-FU (Yuan et al., 2019) or doxorubicin (Thorn et al., 2011) are fewer, meaning it could be considered as a solution for colon cancer therapy.

We tried to find out the effectiveness of combination therapy with 5-FU or doxorubicin used to treat colorectal cancer. OP-D showed the combinational anti-cancer effect of 5-FU or doxorubicin in reducing cell viability and inducing apoptosis through p53 and c-Myc regulation. These results suggest the potential of OP-D for combination therapy with 5-FU or doxorubicin in colon cancer cells.

In summary, our results suggest that OP-D induces apoptosis by activating p53, which requires RPL5 and RPL11, and inhibits c-Myc expression *via* CNOT2 in colorectal cancer as an anti-cancer reagent. These results indicate that OP-D may have uses as a novel therapeutic agent for colorectal cancer patients in the future. Additional experiments are required to support these results, for example exploring whether OP-D regulates p53 expression through MDM2 and the expression of c-Myc *via* ribosomal proteins.

## Data Availability

The original contributions presented in the study are included in the article/Supplementary Material further inquiries can be directed to the corresponding authors.

## Abbreviations

CCK-8, Cell Counting Kit-8; CHX, Cycloheximide; CNOT, CCR4-NOT complex; IPO7, Importin seven; OP-D, Ophiopogonin D; RP, Ribosomal protein; XPO1, Exportin 1.
