# Supplementary figures and images for: Ophiopogonin D increase apoptosis by activating p53 via ribosomal protein L5 and L11 and inhibiting the expression of c-Myc via CNOT2
Source: Front Pharmacol. 2022 Dec 9;13:974468. doi: 10.3389/fphar.2022.974468 (PMC9780504; doi:10.3389/fphar.2022.974468)

Raw data

Fig. 2

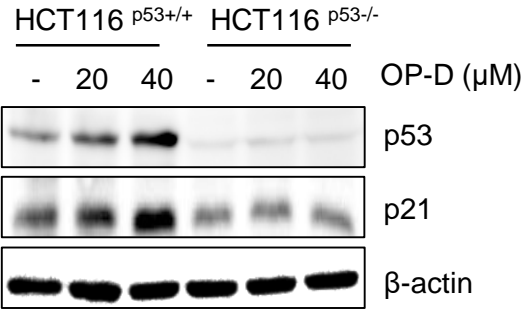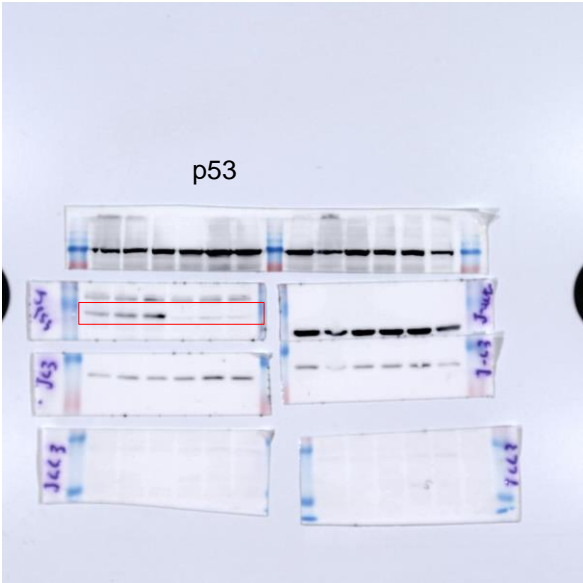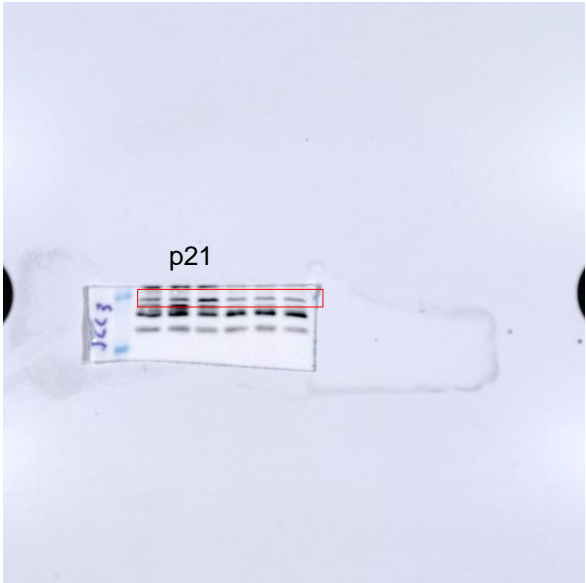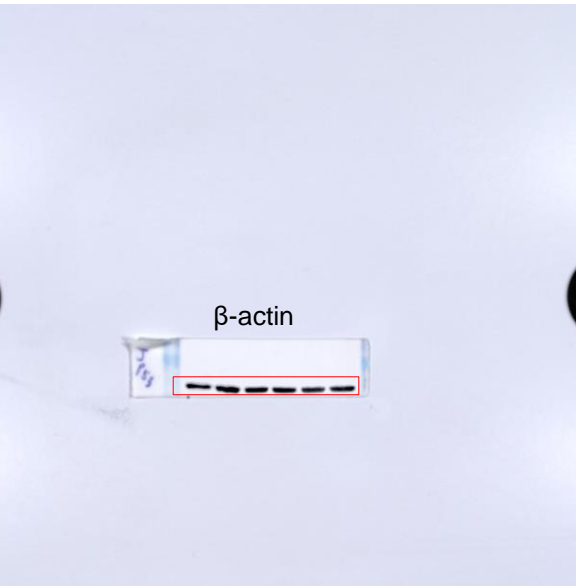

Raw data

Fig. 3

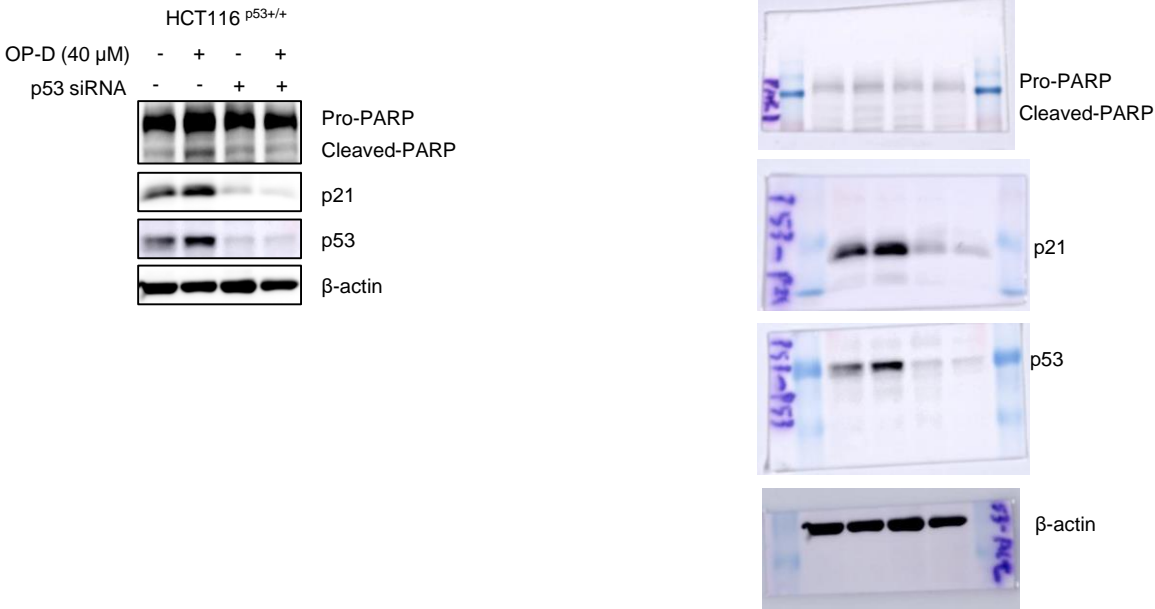

Raw data

Fig. 4

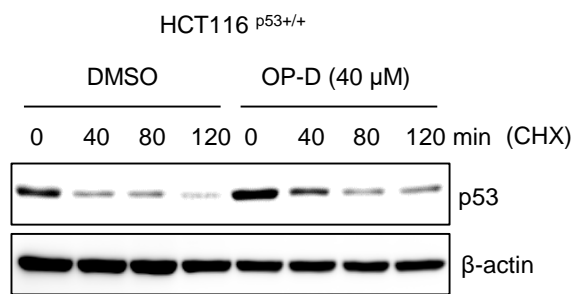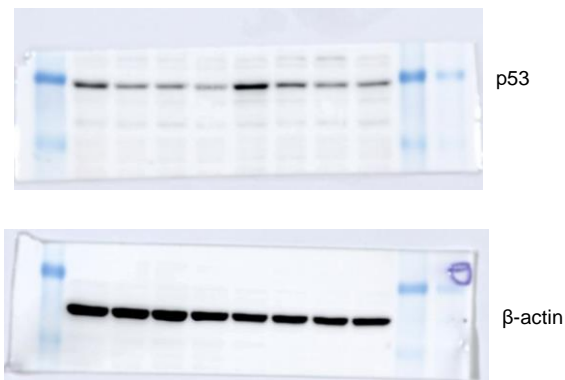

Fig. 5

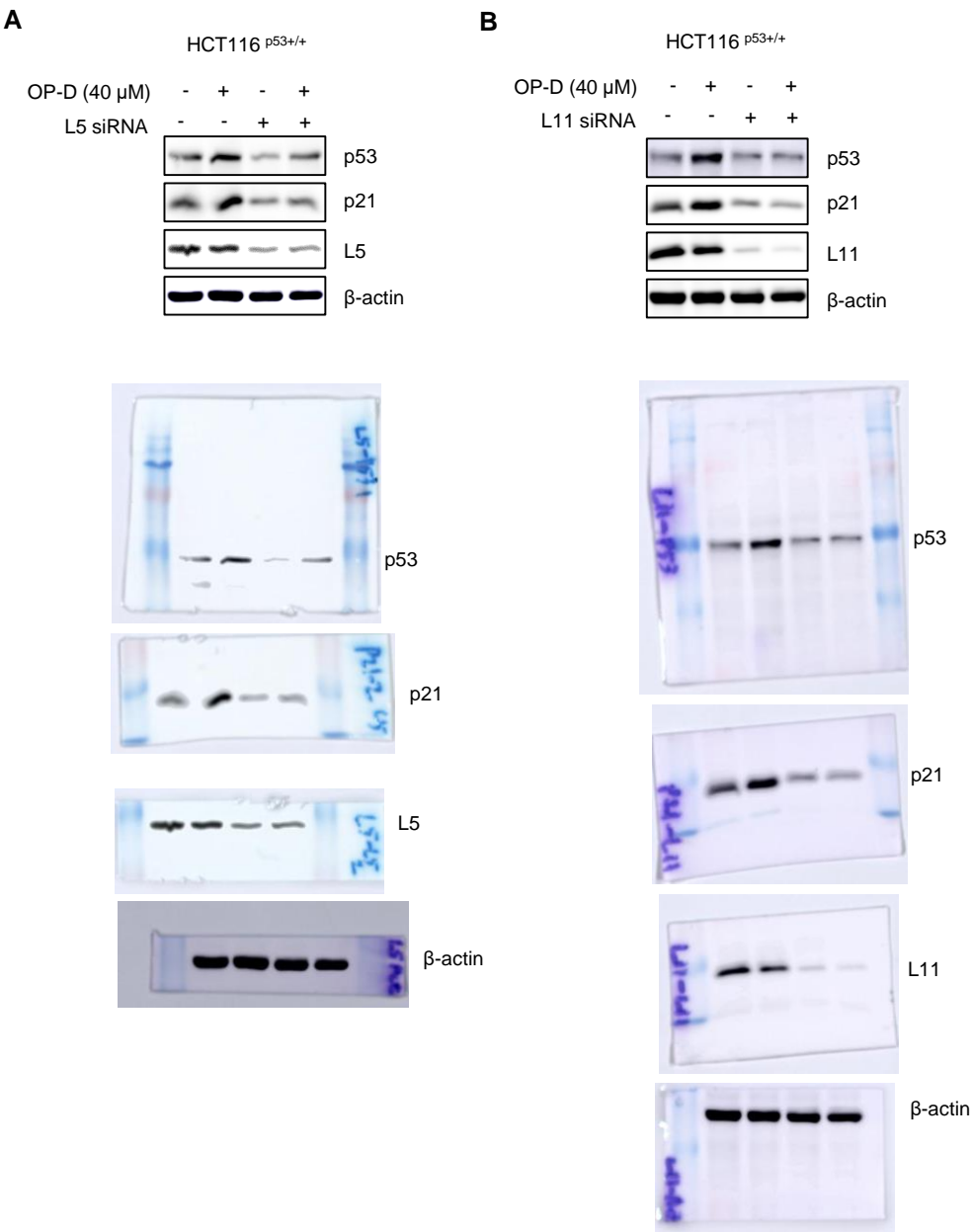

Fig. 6

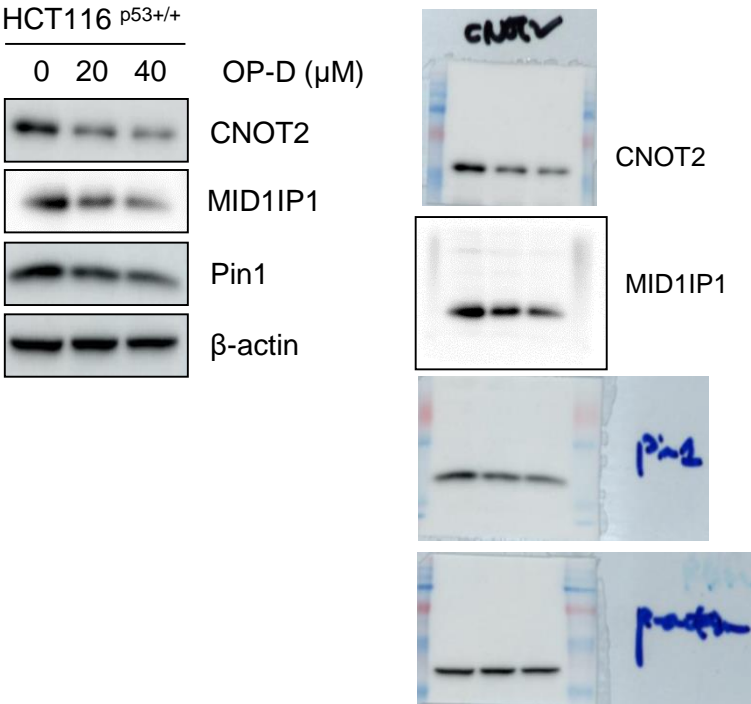

Raw data

Fig. 7

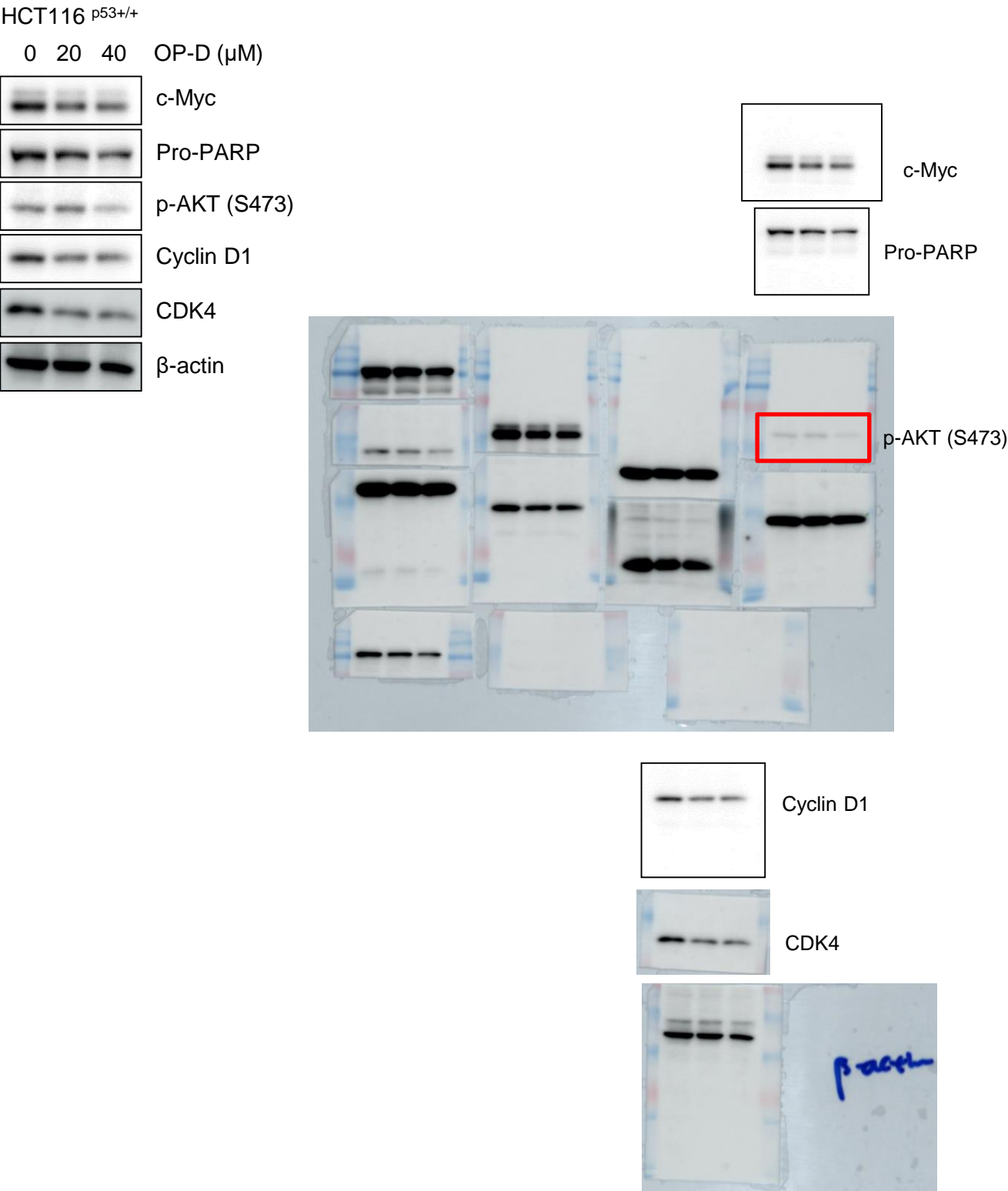

Raw data

Fig. 8

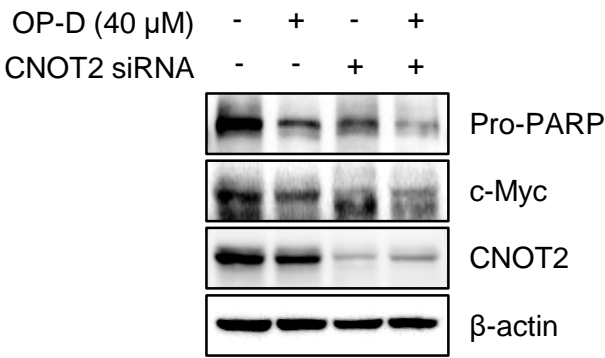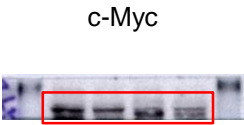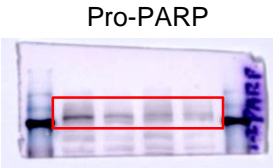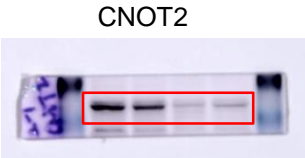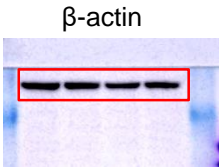

**Fig. 9**

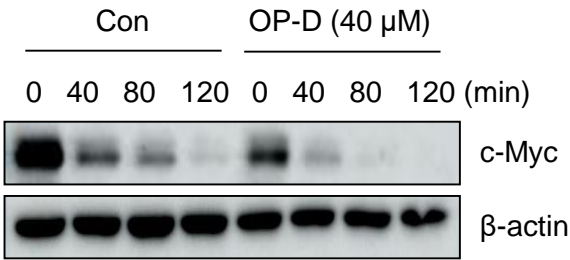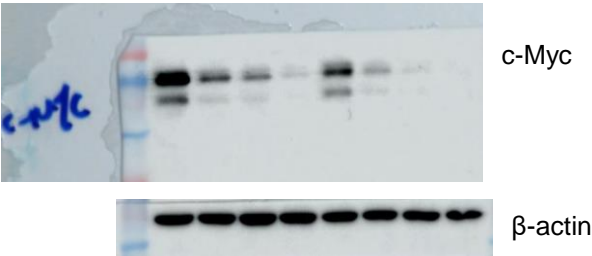

Raw data

Fig. 10C

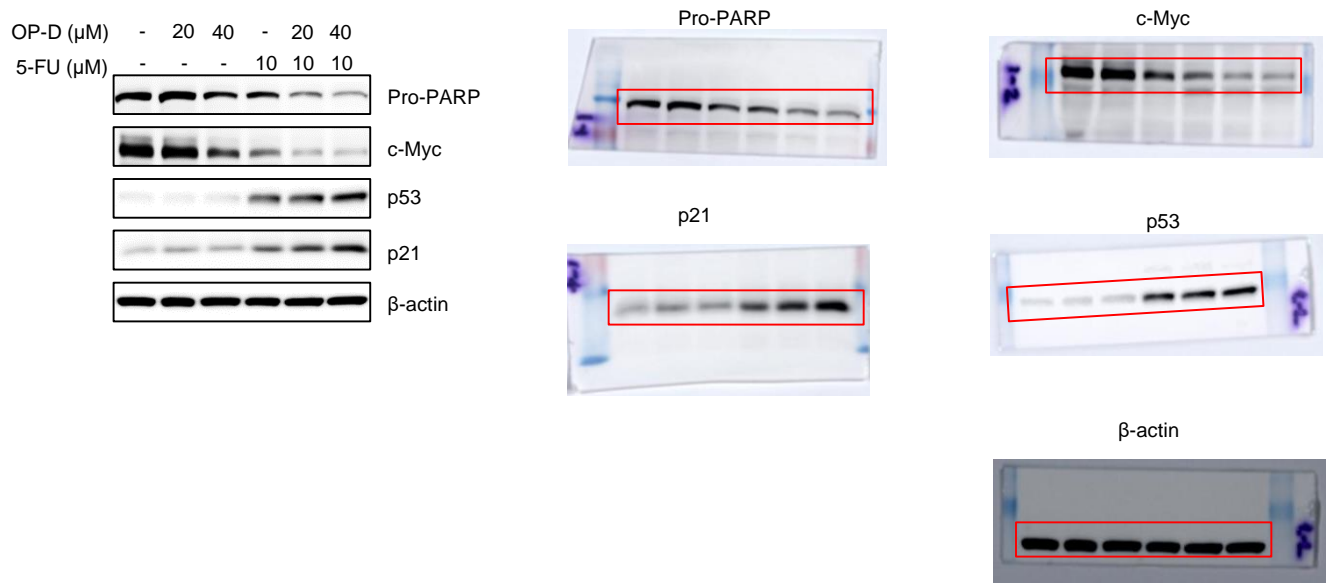

Fig. 10D

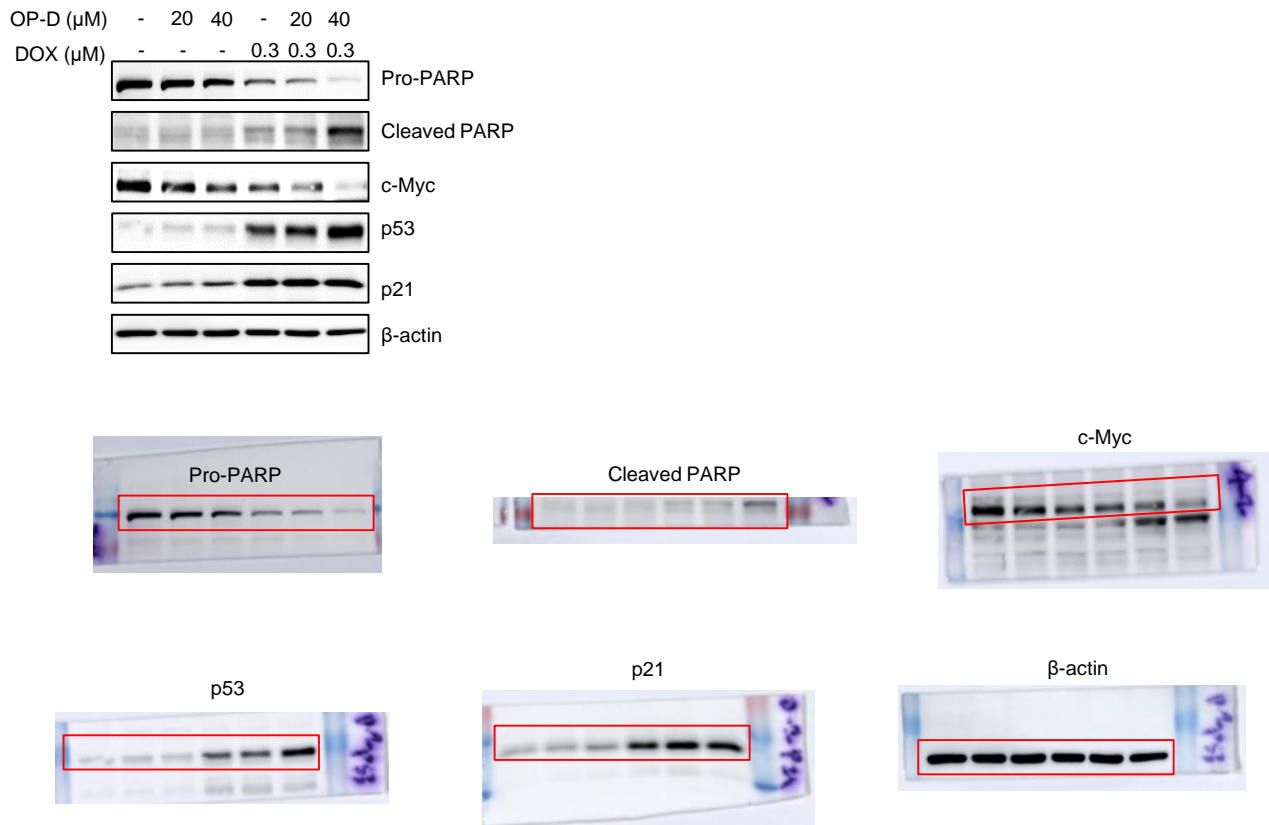

Supplement: Supplementary file 1 [file DataSheet1.PDF]
